# Escape from G1 arrest during acute MEK inhibition drives the acquisition of drug resistance

**DOI:** 10.1093/narcan/zcac032

**Published:** 2022-10-17

**Authors:** Prasanna Channathodiyil, Kieron May, Anne Segonds-Pichon, Paul D Smith, Simon J Cook, Jonathan Houseley

**Affiliations:** Epigenetics Programme, Babraham Institute, Cambridge, CB22 4NT, UK; Epigenetics Programme, Babraham Institute, Cambridge, CB22 4NT, UK; Babraham Bioinformatics, Babraham Institute, Cambridge, CB22 4NT, UK; Oncology R&D, AstraZeneca CRUK Cambridge Institute, Cambridge, CB2 0AA, UK; Signalling Programme, Babraham Institute, Cambridge, CB22 4NT, UK; Epigenetics Programme, Babraham Institute, Cambridge, CB22 4NT, UK

## Abstract

Mutations and gene amplifications that confer drug resistance emerge frequently during chemotherapy, but their mechanism and timing are poorly understood. Here, we investigate *BRAF^V600E^* amplification events that underlie resistance to the MEK inhibitor selumetinib (AZD6244/ARRY-142886) in COLO205 cells, a well-characterized model for reproducible emergence of drug resistance, and show that *BRAF* amplifications acquired *de novo* are the primary cause of resistance. Selumetinib causes long-term G1 arrest accompanied by reduced expression of DNA replication and repair genes, but cells stochastically re-enter the cell cycle during treatment despite continued repression of pERK1/2. Most DNA replication and repair genes are re-expressed as cells enter S and G2; however, mRNAs encoding a subset of factors important for error-free replication and chromosome segregation, including TIPIN, PLK2 and PLK3, remain at low abundance. This suggests that DNA replication following escape from G1 arrest in drug is more error prone and provides a potential explanation for the DNA damage observed under long-term RAF–MEK–ERK1/2 pathway inhibition. To test the hypothesis that escape from G1 arrest in drug promotes *de novo BRAF* amplification, we exploited the combination of palbociclib and selumetinib. Combined treatment with selumetinib and a dose of palbociclib sufficient to reinforce G1 arrest in selumetinib-sensitive cells, but not to impair proliferation of resistant cells, delays the emergence of resistant colonies, meaning that escape from G1 arrest is critical in the formation of resistant clones. Our findings demonstrate that acquisition of MEK inhibitor resistance often occurs through *de novo* gene amplification and can be suppressed by impeding cell cycle entry in drug.

## INTRODUCTION

The development of targeted anti-cancer drugs has improved treatment efficacy and reduced side effects, but drug resistance still limits long-term patient survival (1,2). Mutations and gene amplifications affecting the drug target or proteins in downstream pathways allow re-emergence of tumours that are refractory to treatment with the original and related chemotherapeutics (3,4).

Constitutive activation of the RAS–RAF–MEK–ERK1/2 pathway (hereafter, ERK1/2 pathway), resulting from mutational activation of BRAF or KRAS proteins, occurs in the majority of melanomas and colorectal cancers (5,6). Consequently, the ERK1/2 pathway is a major target for drug development, and inhibitors of RAF and MEK are approved for treatment of melanoma, while ERK1/2 inhibitors are undergoing clinical trials; however, patients often relapse with drug-resistant tumours (7,8). For example, selumetinib (AZD6244/ARRY-142886) is a highly specific MEK inhibitor (MEKi) that suppresses constitutive activity of the ERK1/2 pathway and shows promise in pre-clinical studies (9,10), but resistance to MEKi often arises through amplification of *BRAF* or *KRAS* (11–15).

Cancer cells are genetically heterogeneous, and rare pre-existing mutations that confer drug resistance may be positively selected under drug treatment (16,17). However, acquired mutations that occur during drug exposure can also cause resistance (18), in which case cells must survive initial drug application and then gain mutations that restore proliferation. In culture, and recently *in vivo*, small numbers of drug-tolerant persister (DTP) cells have been observed to survive extended treatment with targeted chemotherapeutics (19–22). DTPs exist in a non-proliferative or slow cycling state with gene expression patterns and metabolic states distinct from untreated and resistant populations (19–21,23–26). However, proliferative colonies routinely emerge from DTPs in the presence of drug after long periods of apparent stasis, marking the DTP state as a precursor to resistance (18,20–22). DTPs do not stem from a genetically defined subpopulation in the parental cell line and are not inherently drug resistant since removal from drug restores normal susceptibility (19,21,22), but colonies of resistant cells derived from DTPs carry drug resistance mutations of unknown provenance and emerge with kinetics consistent with acquired mutation (18,27). Recently, inhibition of EGFR, which acts upstream of the ERK1/2 pathway, was shown to downregulate DNA replication and repair genes while inducing error-prone DNA polymerase genes, which may indicate entry to a mutagenic state in response to drug exposure (28–30).

The cause of *de novo* mutation in DTPs is of great interest as mutagenic mechanisms that act during therapy could be inhibited to slow the acquisition of resistance. Here, we have made use of COLO205 cells treated with the MEKi selumetinib; this is a well-established and reproducible model of tolerance converting to resistance through gene amplification of the addicted oncogene, *BRAF^V600E^* (12,13). We demonstrate that MEKi resistance arises predominantly through *de novo BRAF* amplifications in colorectal cancer cells. Although expression of DNA replication and repair genes is decreased during treatment as previously reported, we find that most are re-expressed as DTPs sporadically enter S phase. However, expression of a subset of genes important for error-free DNA replication remains low throughout the cell cycle in drug, and reducing the frequency of DNA replication events in drug delays the formation of selumetinib-resistant clones. Our results implicate DNA replication in drug as a major driver of *de novo* mutation leading to drug resistance in DTPs.

## RESULTS

### Selumetinib resistance in COLO205 cells arises primarily through *BRAF^V600E^* amplifications acquired *de novo*

Colorectal cancer cells carrying the *BRAF^V600E^* mutation can overcome MEK inhibition by amplification of *BRAF^V600E^*, increasing levels of BRAF^V600E^ protein to activate more MEK and sustain ERK1/2 activity (11,12). However, such *BRAF*^*V600E*^-amplified cells become addicted to MEKi; withdrawal of MEKi drives excessive MEK–ERK1/2 activity due to an over-abundance of BRAF^V600E^, resulting in cell cycle arrest, senescence and apoptosis (12,31). Since a level of *BRAF^V600E^* amplification that is sufficient for resistance should not be tolerated in a drug-naïve cell, *BRAF* amplifications acquired during treatment seem likely to underlie MEKi resistance. However, pre-existing *BRAF*-amplified cells (∼4%) have been reported in drug-naïve colorectal cancer cells (11). These conflicting observations led us to investigate the contributions of pre-existing and acquired *BRAF^V600E^* amplifications to the emergence of MEKi resistance in colorectal cancer cell lines.

**Figure 1.**
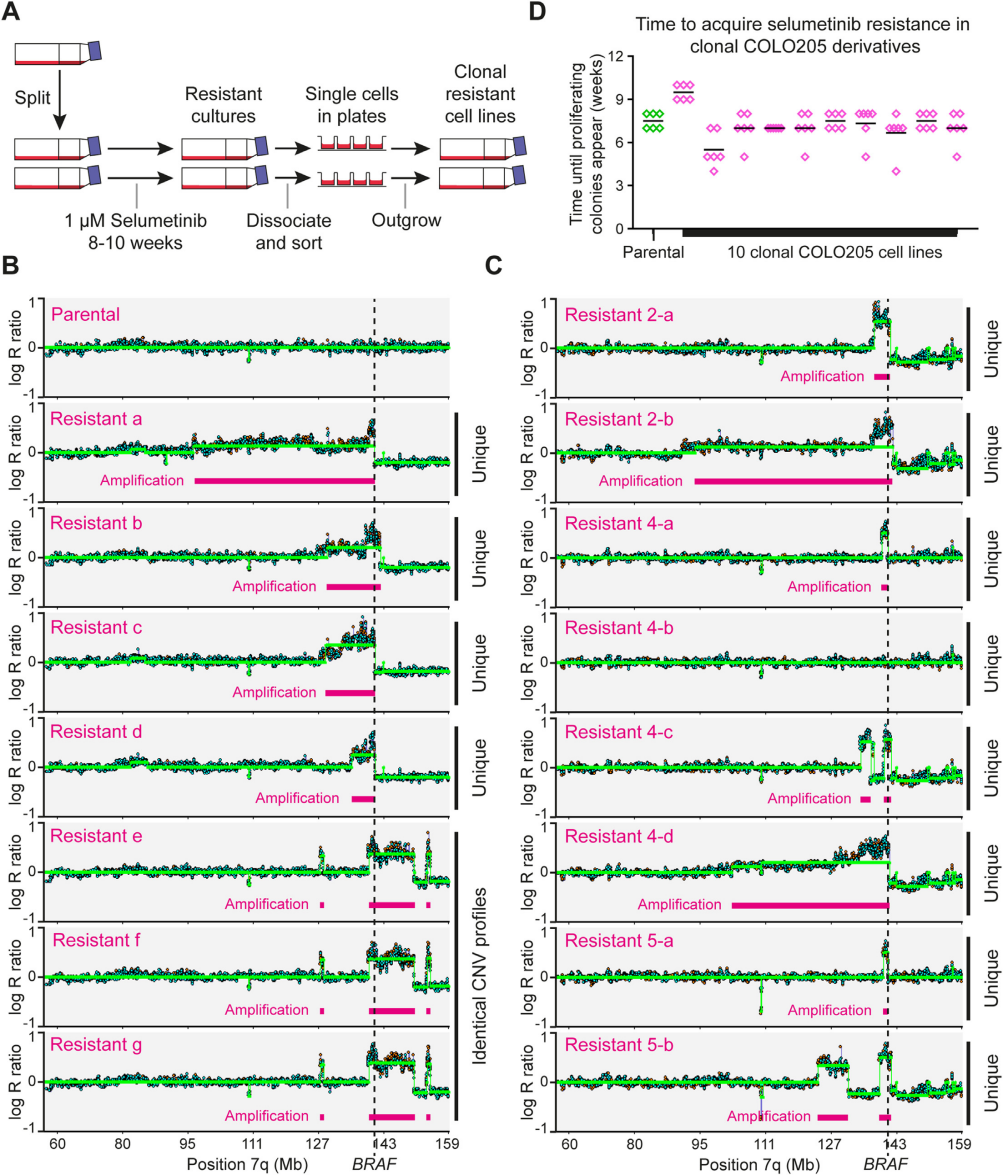
Contributions of pre-existing and *de novo* gene amplifications to the emergence of selumetinib resistance in COLO205 cells. (**A**) Experimental design for analysing reproducibility of resistance. COLO205 cells were cultured in 24 individual 25 cm^2^ cell culture flasks in media containing 1 μM selumetinib. Media and drug were changed weekly until colonies of proliferating cells were observed, at which point single cells were isolated by flow cytometry and expanded into separate drug-resistant cell lines in the presence of 1 μM selumetinib. (**B**) Copy number profiles of the *BRAF* locus in parental COLO205 cells and seven selumetinib-resistant cell lines, determined using CytoSNP-850K BeadChip arrays (Illumina). Log_2_ ratio plots of copy number for the q arm of chromosome 7 are shown, including the location of *BRAF* (dotted line) and amplified regions (pink bars). Each resistant cell line derived from clonal amplification of individual selumetinib-resistant cells from independent drug treatment flasks, but note that cell lines e, f and g show identical and highly characteristic copy number amplification profiles. (**C**) Copy number variation (CNV) profiles of the *BRAF* locus in eight selumetinib-resistant lines obtained from clonal parental cell lines, each resistant cell line derived by clonal amplification from an independent drug treatment flask. Three clonal parental cell lines (clonal cell lines 2, 4 and 5) were used, with four resistant clones derived from cell line 4 and two each from cell lines 2 and 5. Note that all CNV profiles are different, and that cell line 4-b has become resistant without amplification of the *BRAF* locus or any region detectable by array-based CNV analysis. (**D**) Time taken for proliferating selumetinib-resistant clones to emerge from parental and 10 different single cell-derived COLO205 cell lines. For each cell line, cells were seeded in six-well plates and treated individually with 1 μM selumetinib after 24 h. Media and drug were changed weekly, and the time taken to colony formation was recorded (in weeks). The time to resistance was not significantly different between the parental line and any of the clonal lines (*P* > 0.5) by a Kruskal–Wallis test.

Copy number profiling of seven selumetinib-resistant COLO205 clones, derived from seven independent drug-treated cultures, revealed that three clones (Resistants e, f and g) shared identical *BRAF* amplifications that must have been present in the parental cell line prior to drug exposure, while the other four resistant lines (Resistants a–d) carried unique amplicon structures (Figure 1A and B). Sanger sequencing revealed that the *BRAF^V600E^* allele rather than the wild-type allele was amplified in each case (Supplementary Figure S1A). The unique amplicon structures must have either formed *de novo* or emerged from a highly heterogeneous *BRAF*-amplified population in the parental line. To separate these possibilities, we erased existing population heterogeneity by deriving 10 clonal COLO205 cell lines, all of which were sensitive to selumetinib and generated resistant clones by prolonged drug exposure. Copy number profiles of eight resistant cell lines derived independently from three of the clonal COLO205 cell lines revealed seven unique *BRAF* amplicons representing different *de novo* amplification events, and one resistant line with no detectable amplification that must have gained resistance by a different mechanism (Figure 1C). Importantly, the time taken for resistant colony formation in nine of the clonal cell lines was identical to parental COLO205 cells, with the other line being only slightly delayed (Figure 1D). This shows that *BRAF* amplification occurs frequently in COLO205 cells and that pre-existing *BRAF* amplifications in the parental COLO205 cell line contribute little to the timing of selumetinib resistance.

### Individual cells enter the cell cycle even under acute MEK inhibition

**Figure 2.**
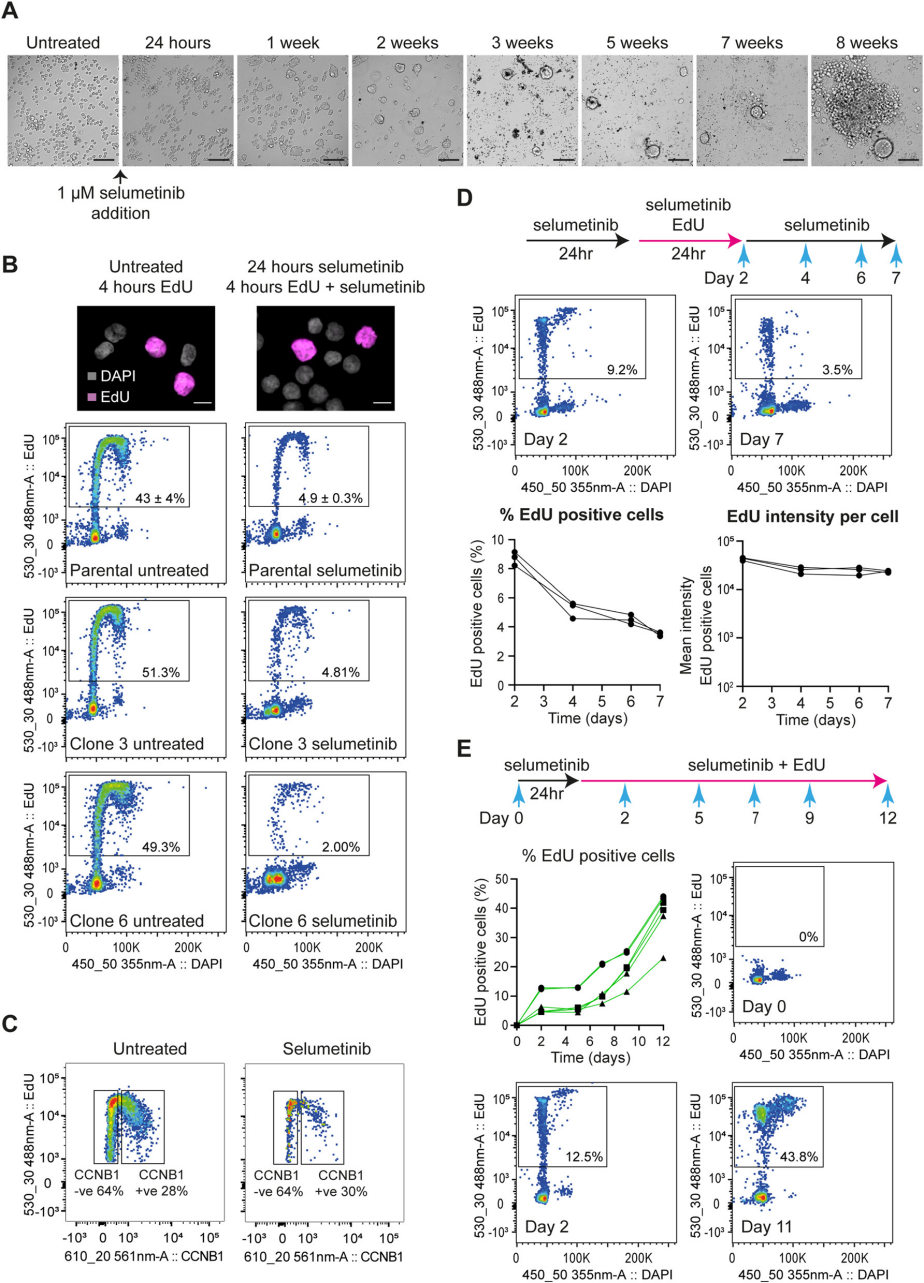
Replicating cells persist in long-term selumetinib-treated cell cultures. (**A**) Representative bright-field images of COLO205 cells during extended treatment with 1 μM selumetinib (scale bars, 100 μm). (**B**) EdU incorporation in COLO205 cells treated for 24 h with 1 μM selumetinib before addition of 10 μM EdU for 4 h. Representative images of EdU-negative and -positive cells (pink) co-stained with DAPI (grey) from selumetinib-treated and control cells are shown at top (scale bars, 10 μm) and quantification of EdU-positive cells in each population by flow cytometry shown below. Percent EdU-positive cells are shown within the gates for each sample; figures for parental line are an average of three experiments with SD. (**C**) Quantification by flow cytometry of CCNB1-negative and -positive cells among the EdU-positive cell population in untreated (left) and selumetinib-treated (right) samples. COLO205 cells were treated with 1 μM selumetinib or DMSO only for 24 h before addition of 10 μM EdU for 4 h in the presence or absence of 1 μM selumetinib, respectively. Cells were stained with EdU reaction cocktail and counterstained with CCNB1 primary antibody and Alexa Fluor 594 conjugated donkey anti-rabbit secondary antibody. Percent CCNB1-positive and -negative cells in the EdU-positive population are shown within the gates for each sample. (**D**) COLO205 cells were treated with 1 μM selumetinib for 24 h before addition of 2 μM EdU for 24 h in the presence of 1 μM selumetinib, after which cells were rinsed in culture media and grown in the presence of 1 μM selumetinib only for up to 5 days. EdU incorporation was assayed at the indicated time points by flow cytometry. Results are mean of two independent replicates. Quantitation of EdU-positive cells (left) and EdU intensity per cell (right), *n* = 3, are shown in the bottom panel. (**E**) Quantification of EdU-positive cells by flow cytometry in COLO205 cells grown in the presence of selumetinib and EdU over the course of 11 days. COLO205 cells were treated for 24 h with 1 μM selumetinib before addition of 2 μM EdU for 6 days, after which cells were rinsed with culture media and then treated with 1 μM selumetinib and 2 μM EdU for a further 5 days. EdU incorporation was assayed at the indicated time points. Data for six independent replicates are shown.

Inactivation of the ERK1/2 pathway by MEK inhibition induces G1 cell cycle arrest in *BRAF^V600E^* cell lines, including COLO205 (32,33). The treatment conditions used here result in growth arrest of COLO205 cells with no passaging required across 6 or more weeks in the presence of drug; instead, gradual cell death occurs over many weeks with remaining DTPs aggregating into large bodies from which resistant colonies often, but not always, emerge (Figure 2A). Since *de novo* gene amplification normally occurs through errors in DNA replication or chromosome segregation (34,35), we assessed whether selumetinib-treated cells escape G1 arrest using incorporation assays for the thymidine analogue ethynyl deoxyuridine (EdU). A 4-h EdU pulse applied 24 h after addition of selumetinib to COLO205 cells labelled 4.9 ± 0.3% of cells, compared to 43 ± 4% of control cells (Figure 2B), confirming that a fraction of cells undergo DNA replication even in the presence of selumetinib. Equivalent results were obtained in clonal COLO205 cell lines (Figure 2B), and EdU-positive cells were detectable at all times analysed up to at least 7 days after selumetinib application (Supplementary Figure S1B). Notably, Ki67, a widely used marker for proliferative cells, is depleted during selumetinib treatment, so the cells do not remain poised for rapid re-entry to the cell cycle and must enter a quasi-G0 state (Supplementary Figure S1C). Therefore, COLO205 cells occasionally enter the cell cycle and initiate DNA replication during extended selumetinib treatment despite the seemingly robust G1 arrest. To ensure that escape from arrest is not unique to COLO205 cells, we analysed another *BRAF^V600E^* mutant colorectal cancer cell line, HT29, and observed a similar proportion of EdU-positive cells during selumetinib treatment (Supplementary Figure S1D). Similarly, we observed replicating cells after treatment of COLO205 cells with the MEKi trametinib, showing that escape from G1 and entry to replication is not unique to selumetinib (Supplementary Figure S1E).

After the 4-h EdU pulse, ∼30% of EdU-positive cells co-stained for the G2 marker Cyclin B1 (CCNB1) in both control and selumetinib-treated populations (Figure 2C), and DAPI incorporation of EdU/CCNB1 double-positive cells was consistent with 4*n* genome content (Supplementary Figure S1F), showing that cells progress through the cell cycle after escaping G1 arrest. The detection of cycling cells during extended selumetinib treatment could be explained by stochastic escape from G1 arrest or continued proliferation of a small subpopulation. To distinguish these, we first performed an EdU pulse-chase experiment and observed that cells labelled during a 1-day EdU pulse did not increase in number or decrease in EdU intensity during the 5-day chase period (Figure 2D). This means that incorporation of EdU by COLO205 cells causes permanent arrest prior to cell division, which we determined to be independent of selumetinib treatment (Supplementary Figure S1G). We then treated cells continuously for 11 days with EdU + selumetinib, during which time almost 50% of cells incorporated EdU (Figure 2E). Given that EdU incorporation causes a permanent arrest without cell division, the progressive increase in EdU-positive cell number over 11 days must reflect new cells entering S phase that have not undergone DNA replication since the addition of EdU, and therefore at least half the population can escape G1 arrest and undergo DNA replication during MEKi treatment. We conclude that the continued presence of cells undergoing DNA replication during prolonged selumetinib treatment results from stochastic escape from G1 arrest among the general population rather than a small proliferating subpopulation.

### Gene expression during cell cycle progression in selumetinib

Suppression of ERK1/2 signalling is reported to downregulate DNA repair genes (30,36–38), and indeed many DNA replication and repair genes were expressed at a significantly lower level in COLO205 cells after 24–48 h of selumetinib treatment (Supplementary Figure S2A). DNA replication without normal expression of replication and repair genes is likely to be mutagenic, but it is unclear whether ERK remains inactive during sporadic re-entry to the cell cycle in drug, or whether these genes remain repressed since replicating cells in drug are too scarce to contribute to bulk mRNA-seq profiles.

**Figure 3.**
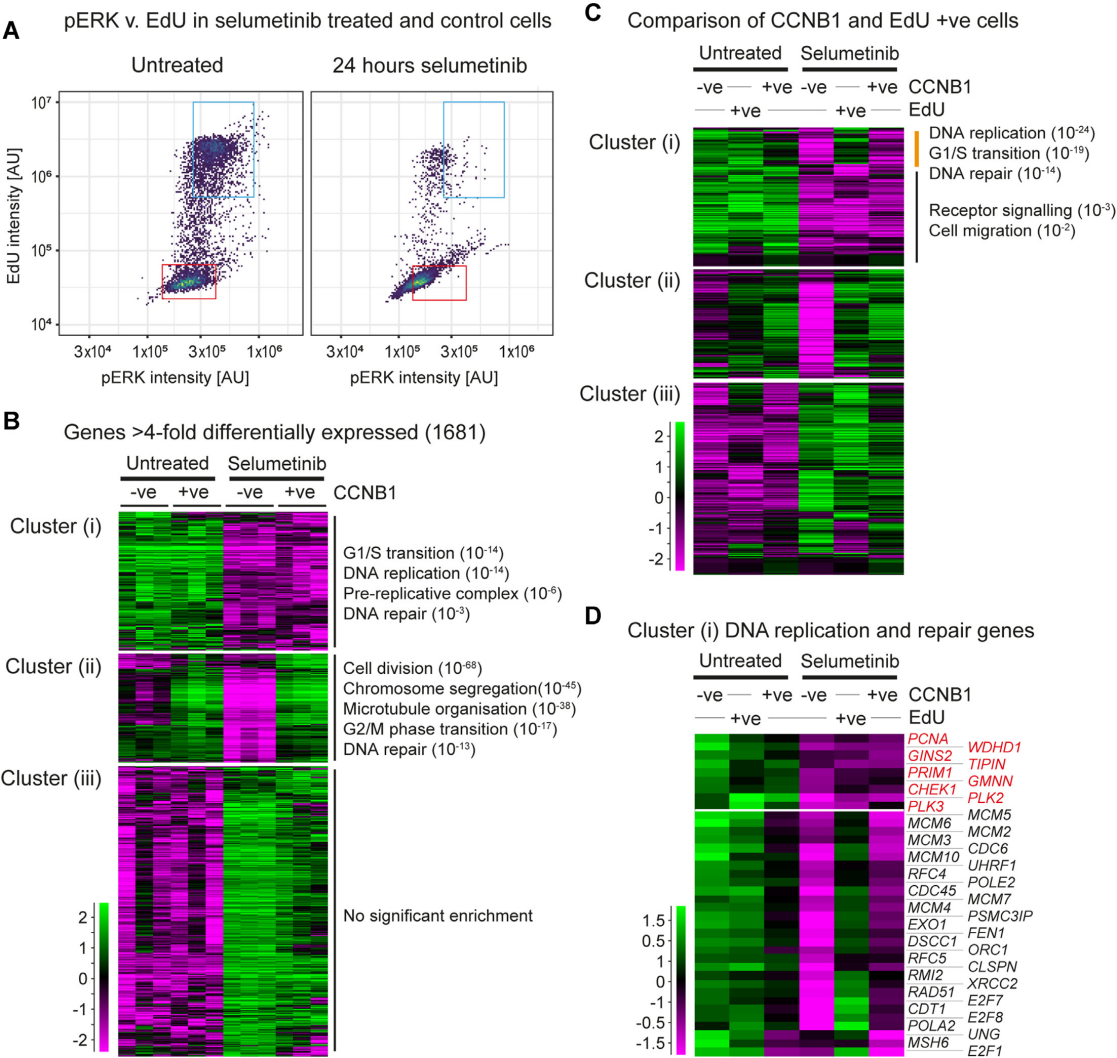
COLO205 cells replicating in selumetinib show defective gene expression. (**A**) Quantification of pERK in EdU-negative and -positive cells. COLO205 cells were treated with 1 μM selumetinib or DMSO only for 24 h before addition of 10 μM EdU for 4 h in the presence of 1 μM selumetinib. Following EdU staining and immunofluorescence with pERK (T202/Y204) antibody, EdU incorporation and pERK levels in cells were determined by high-content image analysis. EdU and pERK intensities in untreated (left) and selumetinib-treated (right) cells normalized to control cells without addition of EdU or pERK primary antibody are shown. EdU-negative and -positive cells in individual plots are shown in red and blue rectangular gates, respectively. (**B**) Differential gene expression between CCNB1-negative and -positive COLO205 cells, either untreated or after 24-h selumetinib treatment. Cells from three biological replicates were fixed with glyoxal, then stained and sorted for CCNB1 followed by mRNA-seq library preparation. The 1681 genes shown are significantly (*P* < 0.05 by DESeq2) and substantially (>4-fold) differentially expressed between at least one pair of the four categories shown. Genes were categorized into three primary behaviours by hierarchical clustering, and representative enriched GO categories (*q* < 0.05) are shown (full GO analysis is presented in Supplementary Table S1). (**C**) Expression of gene clusters (i)–(iii) described in panel (B) in S/G2 cells. Cells were treated with 10 μM EdU for 4 h prior to glyoxal fixation, followed by fluorophore conjugation, sorting and mRNA-seq. Cluster (i) splits into genes transiently upregulated in EdU-positive but not CCNB1-positive cells (expressed in S but not G2), and genes equivalently expressed in EdU- and CCNB1-positive cells; separate GO analyses are shown for these subclusters and full GO analysis is presented in Supplementary Table S2. (**D**) Expression of cluster (i) genes associated with DNA replication in selumetinib-treated and untreated populations sorted for CCNB1 or EdU, extracted from the dataset shown in panel (C).

High-content imaging for phosphorylated ERK1/2 (pERK1/2) showed that ERK is not reactivated in cells replicating in selumetinib, as pERK1/2 was equivalently reduced in EdU-negative and -positive populations under selumetinib treatment (Figure 3A). To study gene expression in rare replicating cells, we developed a method for mRNA-seq after fixation, staining and sorting cells for intracellular markers (39), which we applied to CCNB1-positive G2 cells in selumetinib-treated and control populations (Supplementary Figure S2B). In accord with the pERK1/2 imaging data, CCNB1-positive cells in selumetinib did not re-express genes directly repressed by MEK inhibition (Supplementary Figure S2C, left) (40), nor selumetinib-sensitive targets of the ERK pathway effector RSK (Supplementary Figure S2C, right) (41), nor display the known transcriptomic signature of MEK functional output (Supplementary Figure S2D) (42), so even if replication is initiated by transient ERK1/2 activation this has no lasting impact on the transcriptome. Comparing CCNB1-positive and -negative cells in the presence and absence of selumetinib, 1681 genes were significantly and substantially (>4-fold) differentially expressed between conditions. These formed three hierarchical clusters: (i) genes expressed at a lower level under selumetinib treatment irrespective of cell cycle stage; (ii) genes expressed at a lower level in selumetinib-treated CCNB1-negative cells but expressed at normal levels in CCNB1-positive cells; and (iii) genes expressed at a higher level under selumetinib treatment irrespective of cell cycle (Figure 3B).

Most prominent in cluster (i) genes were GO terms relating to DNA replication, driven by transcripts encoding the entire MCM complex, replicative polymerase epsilon and alpha subunits, and other important replication proteins including PCNA, PRIM1, TIPIN and CLSPN, regulators such as PLK2, PLK3 and GMNN, and repair proteins RAD51 and EXO1. mRNA abundance for all these genes was low in both CCNB1-negative and -positive populations under selumetinib treatment, whereas cluster (ii) transcripts are re-expressed to normal levels in CCNB1-positive cells. GO analysis of this cluster reveals strong enrichments for chromosome segregation and also includes genes for DNA repair factors such as *BRCA2*, *BLM*, *GEN1* and *POLQ*, showing that chromosome segregation and DNA repair genes can be induced as required irrespective of ERK1/2 signalling. Cluster (iii) contained genes with a wide range of functions that were not significantly enriched for any GO category. To ensure that the behaviour of these gene sets is not unique to COLO205, we performed an equivalent experiment in HT29 cells, and observed that most genes in each of the three clusters were similarly affected by selumetinib, and that genes following the same expression patterns were enriched for similar GO categories (Supplementary Figure S2E).

These experiments show that key replication genes are mis-expressed in cells escaping selumetinib-induced G1 arrest, but profiling CCNB1-positive cells would miss a transient upregulation of transcripts during S phase. RNA recovered from EdU-treated cells is inevitably degraded during click labelling of EdU, but we were able to quantify transcript 3′ ends from EdU-positive cell samples (Supplementary Figure S2F). Reassuringly, cyclin mRNAs followed expected distributions: *CCNE1* and *CCNE2* were high in EdU-positive cells, *CCNB1* was high in CCNB1-positive cells and *CCND1* was high in both though only in the absence of selumetinib (Supplementary Figure S2G). Across the three clusters defined above, the profiles of EdU-positive cells were similar to CCNB1-positive cells, but showed induction of some cluster (i) genes (Figure 3C, orange bar). These genes were highly enriched for DNA replication and DNA repair categories and included the *MCM* genes, replicative polymerase subunits, *CLSPN*, *RAD51* and *EXO1*, showing that most key replication and repair genes are induced on sporadic entry to S phase during selumetinib treatment. However, the expression of *PCNA*, *WDHD1*, *PRIM1*, *TIPIN*, *PLK2*, *PLK3*, *CHEK1* and *GMNN* remained low across G1, S and G2 under MEKi treatment (Figure 3D). Depletion of any of these genes decreases genome stability (43–48), providing support for the idea that DNA replication and chromosome segregation in MEKi-treated cells will be more error prone.

### Escape from G1 arrest during MEK inhibition facilitates the emergence of drug resistance

**Figure 4.**
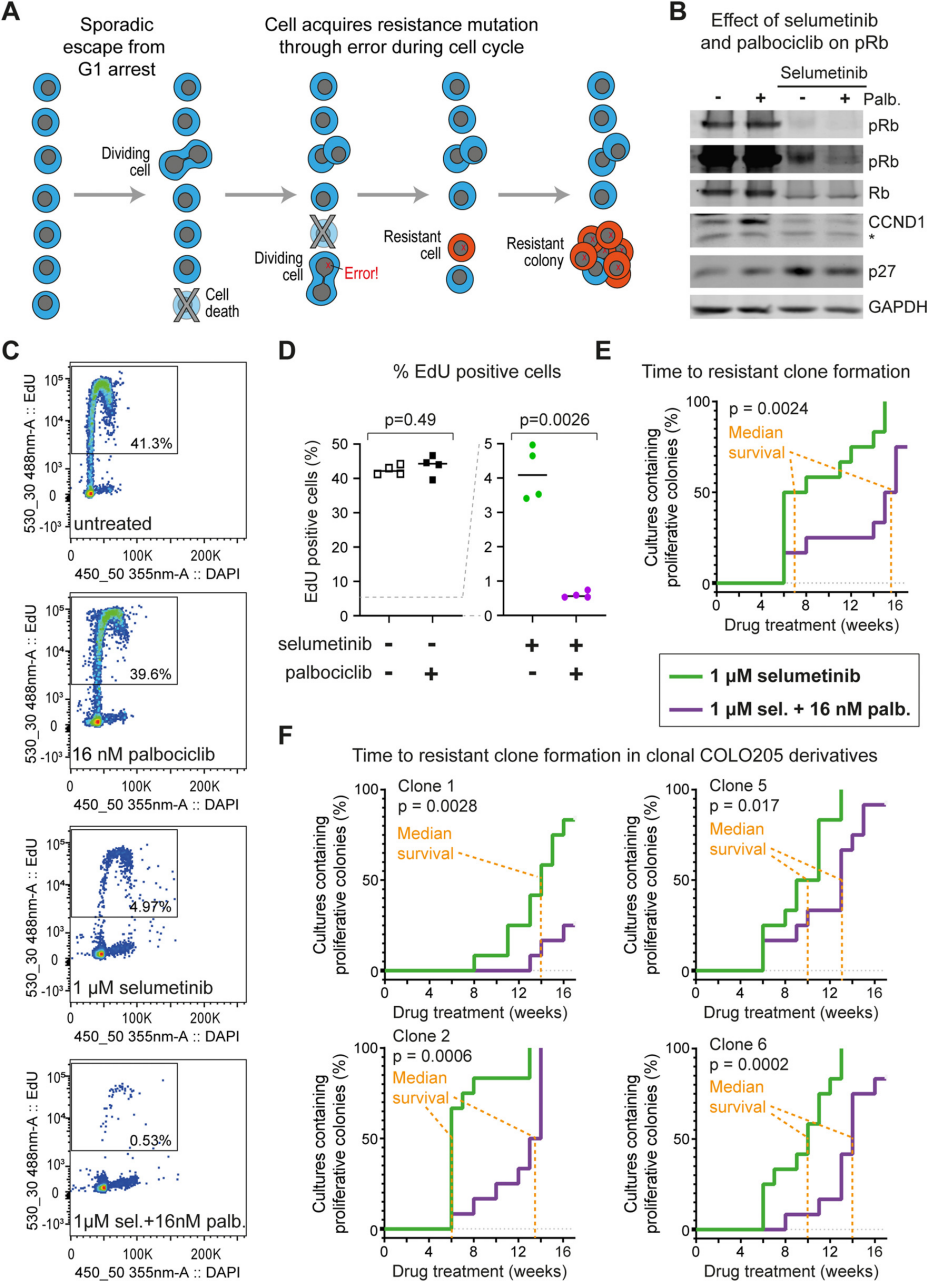
Suppressing DNA replication in selumetinib slows acquisition of resistance. (**A**) Proposed mechanism for emergence of *de novo* resistance in drug. Selumetinib-treated cells are arrested in G1 because of ERK1/2 pathway inhibition. Occasionally, cells escape the G1 arrest and undergo a cell cycle, but this slow proliferation is offset by ongoing cell death and is not detectable in the bulk population. Occasional cell cycle events result in DNA replication or chromosome segregation errors that give rise to *de novo* mutations, some of which bestow drug resistance. This manifests as the sudden appearance of a rapidly proliferating colony after a long period of apparent stasis. (**B**) Western blot analysis of COLO205 cells treated with 1 μM selumetinib in the presence (+) or absence (−) of 16 nM palbociclib for 24 h and with the indicated antibodies. The pRB panels are shown at two different intensities to make the reduction of pRB levels in the combined selumetinib and palbociclib condition visible. Other panels show total Rb, CCND1, p27 (which is also part of the active CCND1–CDK4/6 complex) and GAPDH as a loading control. * indicates non-specific band. (**C**) Quantification of EdU-positive cells by flow cytometry in COLO205 cells treated with palbociclib and/or selumetinib at the indicated concentrations for 24 h before addition of 10 μM EdU for 4 h. Percent EdU-positive cells are shown within the gates for each sample. (**D**) Quantification of EdU-positive cells in panel (C). *n* = 4 (two biological replicates each of parental COLO205 cells and single cell-derived clone 2); *P*-values calculated by *t* test with Welch’s correction. (**E**) Effect of combined treatment with selumetinib and palbociclib on time taken for the emergence of resistant clones in parental COLO205 cell line. Cells were cultured in media containing 1 μM selumetinib in the absence or presence of 16 nM palbociclib. Media and drug were replenished weekly, and the time taken for the appearance of first colony (≥50 cells) of proliferating cells in each well was recorded (in weeks). Twelve independent replicates were performed under each condition; *P*-values were calculated using the Mantel–Cox log-rank test on the principle that emergence of resistance can be represented as survival time of non-resistant cultures. (**F**) Effect of combined treatment with 1 μM selumetinib and 16 nM palbociclib in single-cell derivatives of COLO205 cells determined as in panel (E). Data for four different single-cell derivatives of COLO205 are shown.

Disrupted expression of *TIPIN*, *PLK2* or *PRIM1* increases replicative stress and reliance on ATR signalling (46,49,50) and indeed selumetinib-treated COLO205 cells are significantly more sensitive to the ATRi AZ20 (Supplementary Figure S3A, right), though the effect is small since replicating cells that would be sensitive to ATRi are rare in selumetinib-treated populations. Replication stress caused by disrupted gene expression would explain reports of γH2AX-positive cells during MEKi treatment (30,51), but this transient replication stress would be alleviated by the return to apparently normal proliferation in resistant lines, and indeed we do not detect elevated γH2AX in selumetinib-resistant COLO205 clones (Supplementary Figure S3B). Replicative stress increases the frequency of errors in DNA replication and chromosome segregation [reviewed in (52)], raising the hypothesis that replicative stress in the cell cycle following sporadic escape from G1 arrest in drug would engender new mutations and structural rearrangements. It follows that acquisition of resistance mutations such as *BRAF* amplification may arise particularly in cells that escape arrest during drug treatment (Figure 4A).

The link between escape from G1 and acquired mutation is supported by our observation of large CNVs in single-cell sequencing data from COLO205 cells after 48 h selumetinib treatment. Four amplification events and five deletion events, each of two or more copies and >10 Mb in length, were detected among 7 Cyclin B1-positive cells out of 42 tested, whereas no major events were detected in 43 Cyclin B1-negative cells from the same population (*P* = 0.0055 by Fisher’s exact test), consistent with our hypothesis that CNVs arise through cell cycling under selumetinib treatment (Supplementary Figure S3C and D).

If escape from G1 is critical in mediating *de novo* CNV events, then selectively reducing the frequency at which cells escape from selumetinib-mediated G1 arrest, without impairing the proliferative capacity of cells that acquire resistance, should reveal the importance of DNA replication in drug for *de novo* acquisition of resistance mutations. The CCND1–CDK4/6 complex controls exit from G1, and MEKi–CDK4/6i combinations inhibit proliferation more effectively than either inhibitor alone (53–55). We therefore asked whether combining selumetinib with a low dose of CDK4/6i would inhibit the escape of selumetinib-sensitive cells from G1 arrest in selumetinib without impairing cell proliferation (either of selumetinib-sensitive cells in the absence of selumetinib or of selumetinib-resistant cells in the presence of selumetinib).

We determined that treatment of COLO205 cells with the CDK4/6i palbociclib at 16 nM [∼10% IC_50_ (56)] did not reduce proliferation or colony formation in the absence of selumetinib (Supplementary Figure S4A and B), nor impair proliferation of selumetinib-resistant COLO205 cells in the presence of selumetinib (Supplementary Figure S4C). Rb phosphorylation was unaffected by 16 nM palbociclib alone, but residual Rb phosphorylation detected during selumetinib treatment was decreased by addition of 16 nM palbociclib (Figure 4B). EdU incorporation assays confirmed that 16 nM palbociclib had no impact on entry to the cell cycle in the absence of selumetinib but reduced the fraction of EdU-positive cells in the presence of selumetinib by 10-fold (Figure 4C and D). Together, these data show that 16 nM palbociclib alone does not cause G1 arrest, but substantially enhances the G1 arrest mediated by selumetinib, and as 16 nM palbociclib does not affect proliferation in the presence of selumetinib once cells have acquired selumetinib resistance, any effect of palbociclib should arise through effects on G1 arrest in selumetinib prior to the acquisition of resistance.

We then compared the time taken for proliferating drug-resistant colonies to form in cultures treated with selumetinib alone or with a combination of selumetinib and 16 nM palbociclib, in the parental COLO205 cell line and four single cell-derived clones. Resistant colonies formed in 86% of cultures; however, addition of 16 nM palbociclib delayed the appearance of resistant clones substantially and significantly in all five cell lines, extending the median time to resistance by 3–8 weeks (Figure 4E and F). We used a Cox proportional-hazards model to quantify the overall effect of 16 nM palbociclib in combination with selumetinib compared to selumetinib alone, and found that 16 nM palbociclib reduced the risk of resistance by 78% with *P* = 1.6 × 10^−11^. Differences between the COLO205 cell lines had no significant effect on palbociclib action (*P* = 0.93), even though we observed a significant difference between the cell lines in acquisition of resistance in general (*P* = 1.1 × 10^−10^). For example, clone 1 was slow to obtain resistance here as in Figure 1D (*P* = 5.5 × 10^6^) but took even longer to develop resistance under combined treatment with palbociclib.

Mean BRAF amplifications were equivalent for resistant cell lines derived under both conditions, which we expected since the addition of palbociclib is predicted to reduce the frequency at which *BRAF* amplifications form without affecting the magnitude of amplification (Supplementary Figure S4D). However, closer examination revealed that ∼25% of the resistant clones formed during selumetinib + palbociclib treatment underwent minimal *BRAF* amplifications (<2-fold) including some that hardly differ from the parental, whereas all the resistant clones that arose on selumetinib alone carried >2-fold amplifications (Supplementary Figure S4D). This suggests that as the acquisition of *BRAF* amplification is retarded through suppression of escape from G1 arrest by palbociclib, other mechanisms leading to different resistance mutations become more prevalent.

This experiment shows that reducing the frequency of DNA replication during selumetinib treatment delays the emergence of resistant clones, demonstrating that escape from G1 arrest in drug is critical for the acquisition of *de novo BRAF* amplifications. Although the combinatorial suppression of proliferation by MEKi and CDK4/6i is well characterized (53–55), our experiments show an additional beneficial impact of this combination in delaying the acquisition of resistance mutations.

## DISCUSSION

Here, using BRAF^V600E^ mutant colorectal cancer cells we show that *de novo BRAF* amplifications are acquired in selumetinib-treated populations with remarkable efficiency. Cells under continuous selumetinib exposure stochastically escape G1 arrest and enter S phase but do so without inducing a subset of genes encoding factors important for error-free DNA replication and chromosome segregation. Escape from G1 arrest is vulnerable to otherwise inert doses of CDK4/6i such that a MEKi + CDK4/6i combination suppresses DNA replication during selumetinib treatment, thereby retarding the formation of resistant clones.

Reduced expression of high-fidelity DNA replication and repair genes has been observed in drug-arrested cell populations (28,30), which suggests that the sporadic entry of drug-treated cells into S phase may occur when replication factors are limited. However, our analysis of gene expression at specific cell cycle stages shows that DNA replication and repair genes are almost all induced as needed under MEKi treatment, so changes measured by bulk mRNA-seq largely reflect shifts in cell cycle distribution of the population. Nonetheless, a small number of genes important for accurate DNA replication and chromosome segregation are repressed across G1/S/G2 during MEKi treatment, which could underlie an increase in mutagenicity, and indeed our findings link the emergence of *de novo* resistance to cell cycle entry in drug.

One puzzling feature of DTP cells that survive for long periods in the presence of chemotherapeutics such as selumetinib is the sharp transition between drug tolerance and proliferation. The bulk population does not slowly re-acquire the ability to proliferate in drug; instead, individual colonies of rapidly dividing cells suddenly appear after weeks or months of apparent stasis, requiring a marked return to proliferation in a very small number of cells (19–22). The mechanism we propose explains this property (Figure 4A); occasional cell division events would not be noticeable in long-term drug-treated cultures as these are offset by ongoing cell death (some of which may well arise through inappropriate entry to the cell cycle). However, if each replication event carries a risk of *de novo* gene amplification, then each cell has a chance of acquiring the correct amplification to allow proliferation during a sporadic replication event. Gene amplifications arising in this manner would manifest as a sudden return of a single cell to proliferation, with an average time to resistance defined by the frequency of DNA replication events in drug and the extent to which drug treatment reduces the fidelity of replication.

Our findings indicate that resistance to MEKi is often acquired through a defined mechanism that can be inhibited. It is conceivable that a high frequency of cycling tumour cells in a biopsy taken early in treatment, using, for example, Cyclin B1 as a marker, would predict an increased likelihood of resistance acquisition through this mechanism. We suggest that addition of palbociclib to the treatment regimen at this point would be beneficial, and we note that the concentration of palbociclib we applied in culture (16 nM) is well within the clinically achievable range [*C*_max_ = 101 nM (57)]. Palbociclib exerts high selectivity on target kinases inducing robust inhibition of cell cycle progression that does not seem to reflect an off-target effect (58), and treatment with palbociclib is associated with minimal adverse effects compared to other more potent FDA-approved CDK4/6 inhibitors, in particular abemaciclib (59,60).

Mutability under stress is well characterized in bacteria and has been repeatedly observed in yeast (61–64). However, it is hard to prove that such events result from defined programmes that have emerged through selective evolution, against the null hypothesis that mutagenesis is an emergent property of normal maintenance and proliferation systems becoming compromised under stress. We would therefore hesitate to label genome instability caused by under-expression of replication proteins as a mutagenic response, though our study provides strong support for the suggestion that non-genotoxic drug treatment can increase mutation rate and drive the emergence of resistance. Whether mutagenesis is intentional or not, our study and others addressing drug-induced mutation (28,65) provide grounds for optimism that resistance to targeted chemotherapeutics is preventable, since mutational mechanisms that act during chemotherapy can be characterized and suppressed.

Overall, our study shows that pathways to acquired resistance can be mechanistically defined and present vulnerabilities that can be specifically targeted to slow or stop the acquisition of drug resistance.

## MATERIALS AND METHODS

### Cell culture and drug treatment

COLO205 and HT29 cell lines were provided by the laboratory of Dr Simon J. Cook (Babraham Institute). Cells were cultured in RPMI 1640 (COLO205) or McCoy’s 5A (HT29) media supplemented with 10% (v/v) foetal bovine serum, penicillin (100 U/ml), streptomycin (100 mg/ml) and 2 mM glutamine at 37°C in a humidified incubator with 5% (v/v) CO_2_. Selumetinib- and/or palbociclib (Selleckchem)-resistant derivatives were generated by culturing cells in indicated drug concentrations with media and drug replenished weekly until proliferating colonies formed in culture. To generate single-cell derivatives, cells (5 × 10^6^ cells/ml) were incubated with 1 μg/ml DAPI (Sigma) and DAPI-negative cells sorted into 96-well plates containing media on a BD FACSAria III sorter (BD Biosciences). Cell line identity was validated based on RNA-seq data generated in this work using Cell Line Sleuth, developed by Simon Andrews of the Babraham Institute Bioinformatics Facility (https://github.com/s-andrews/celllinesleuth).

### EdU staining and immunofluorescence for imaging

Cells were fixed in 4% formaldehyde and permeabilized in 0.5% Triton X-100 before incubation in a reaction cocktail [43 μl component D, 387 μl water, 20 μl CuSO_4_, 50 μl reaction buffer additive (43 μl 10× reaction buffer additive + 387 μl water) and 1.2 μl Alexa Fluor 594 dye] (Thermo Fisher Scientific) for 30 min at room temperature (RT) in dark and mounted in mounting medium with DAPI (Vector Laboratories). For high-throughput imaging, cells cultured in 96-well plates (Perkin-Elmer) were formaldehyde-fixed, permeabilized in ice-cold 100% methanol for 10 min at −20°C and labelled using an Alexa Fluor™ 647 HCS assay kit (Thermo Fisher Scientific). In both cases, cells were blocked in 5% normal goat serum and 2% BSA for 1 h, followed by incubation in primary antibody at 4°C overnight and secondary antibody for 1 h at RT in dark. Cells were counterstained in DAPI and imaged using an IN Cell Analyzer 6000 Microscope. Details of antibodies are provided in Supplementary Table S5.

### EdU staining and immunolabelling for flow cytometry

EdU labelling was performed using a Click-iT™ EdU Alexa Fluor™ 488 Flow Cytometry Kit (Thermo Fisher Scientific) following the manufacturer’s instructions. EdU reaction mixture consisted of 219 μl PBS, 5 μl CuSO_4_, 25 μl 1× buffer additive and 1.25 μl Alexa Fluor dye. Cells were counterstained in DAPI and analysed on a Fortessa (BD Biosciences) flow cytometer. To isolate cells by flow cytometry following CCNB1 staining, cells were processed as previously described (39). For sorting on EdU, cells were incubated in a modified reaction cocktail [209 μl PBS, 5 μl CuSO_4_, 25 μl 1 M l-ascorbic acid (Sigma, A2174), 1.25 μl Alexa Fluor 488 dye, 10 μl RNasin Plus] (Thermo Fisher Scientific) and incubated on ice for 30 min in dark.

### RNA extraction and mRNA-seq library preparation

RNA was extracted from cells using TRIreagent (Sigma) following manufacturer’s instructions and RNA integrity assessed using a Bioanalyzer 6000 pico chip (Agilent). mRNA-seq libraries were prepared using the NEBNext Ultra (or Ultra II) Directional RNA Kit (NEB) with the NEBNext Poly(A) mRNA Magnetic Isolation Module and processed for sequencing as previously described in (39).

### mRNA-seq data analysis

After adapter and quality trimming using Trim Galore (v0.5.0), RNA-seq data were mapped to human genome GRCh38 using HISAT2 v2.1.0 (66) by the Babraham Institute Bioinformatics Facility. Mapped data were imported into SeqMonk v1.47.0 (https://www.bioinformatics.babraham.ac.uk/projects/seqmonk/) and normalized to total read count. DESeq2 analyses (67) was performed within SeqMonk using a *P*-value cut-off of 0.01, and significantly different genes were further filtered for genes with >4-fold difference in at least one comparison. RNA obtained from EdU-treated cells was of poor quality as the click reaction conditions cause some RNA degradation, so for comparisons involving these datasets (Figure 3C and D) all datasets involved were treated as follows: (i) Reads were filtered and all reads outside an annotated exon were discarded. (ii) The GRCh38 annotation was parsed to yield annotations covering only the 3′ 500 nucleotides only for high-confidence transcripts using the code at https://github.com/s-andrews/three_prime_gtf, and opposite strand reads mapping to this annotation set were quantified. Restricting analysis to the 3′ end reduces the impact of RNA degradation as the RNA-seq library preparation includes a poly(A) selection and therefore fragmented transcripts have a 3′ end bias. (iii) An enrichment normalization to the 50th and 90th percentiles was performed in SeqMonk to match the distributions of the datasets, which minimizes biases resulting from the differences in RNA quality. Hierarchical clustering analysis was performed using SeqMonk, and GO analysis of individual clusters was performed using GOrilla (http://cbl-gorilla.cs.technion.ac.il/) (68,69). Quoted *P*-values for GO analysis are FDR-corrected according to the Benjamini and Hochberg method (*q*-values from the GOrilla output); for brevity, only the order of magnitude rather than the full *q*-value is given (70). GEO accession: GSE168604.

### DNA extraction and qPCR

Genomic DNA from cells was extracted using a DNeasy Blood and Tissue Kit (Qiagen), digested with EcoRI and purified using a QIAquick PCR purification kit (Qiagen). For each qPCR reaction, 4 μl DNA was mixed with 5 μl 2× Maxima SYBR mix, 0.2 μl each of forward and reverse primers (10 μM) (Supplementary Table S6) and 0.6 μl water. Cycling conditions were as follows: 95°C for 10 min and then 40 cycles of 95°C for 15 s and 60°C for 1 min.

### CNV microarray

DNA samples were processed by Cambridge Genomic Services (Cambridge University) for hybridization onto CytoSNP-850K BeadChips (Illumina) following the manufacturer’s instructions. Data were analysed with BlueFuse Multi software version 4.5 and the BlueFuse algorithm with default settings (10 contiguous markers for CNV and 500 contiguous markers for loss of heterozygosity) and mapped to genome build 37. The cluster and manifest files for processing CytoSNP-850K v1.1 were CytoSNP-850Kv1-1_iScan_C1_ClusterFile.egt and CytoSNP-850Kv1-1_iScan_C1.bpm, respectively, and CytoSNP-850Kv1-2_iScan_B1_ClusterFile.egt and CytoSNP-850Kv1-2_iScan_B3.bpm, respectively, for v1.2 beadchips. GEO accession: GSE168604.

### Single-cell bisulfide sequencing

Cells were glyoxal-fixed and stained for CCNB1 as previously described (39) and single cells sorted by flow cytometry to collect CCNB1-positive and -negative cells in a 96-well plate. Single-cell bisulfite libraries were then prepared as previously described (71). Briefly, bisulfite conversion was carried out using EZ Methylation Direct bisulfite reagent (Zymo) and desulfonation and purification of converted DNA with magnetic beads (Zymo) on the Bravo automated workstation (Agilent) with DNA eluted into the master mix for the first-strand synthesis. Random priming and extension were performed followed by purification of the resulting DNA fragments. Second-strand synthesis was then performed and the resulting fragments amplified with 14 PCR cycles. Amplified libraries were pooled (two pools of 48 libraries each) and purified using 0.7× AMPure XP beads. Libraries were assessed for quality and quantity using high-sensitivity DNA chips on the Agilent Bioanalyzer. Libraries were sequenced on a NextSeq500 High Output on 75-bp single-end mode. After adapter and quality trimming using Trim Galore (v0.6.2), they were mapped to GRCh38 using Bismark v0.22.1. Mapped reads were imported into SeqMonk v1.47.0 (https://www.bioinformatics.babraham.ac.uk/projects/seqmonk/). After QC to remove libraries with very low read counts or aberrant distributions, we were left with 44 Cyclin B1-negative and 43 Cyclin B1-positive cells. For these, reads were quantified in 5 Mb windows spaced every 2.5 Mb across the genome, with regions of aberrantly high and low read counts excluded (removes centromeres and other low-complexity regions). Log transformed datasets were normalized per probe by subtracting the median for that probe, which converts data to log_2_ fold change in copy number relative to average (the average copy number for each probe being the genomic copy number). Enrichment normalization for the 10th and 90th percentiles was then applied to each dataset to normalize differences in total read count and distribution.

### Protein extraction and western blot

Preparation of cell lysates for SDS-PAGE and western blotting were performed as previously described (31). Total protein was subjected to electrophoresis through a 10% SDS-PAGE gel for 4 h at 75 V and transferred to methanol-activated Immobilon-FL polyvinylidene difluoride membranes (Merck Millipore) (Figure 4B), or through a 14% SDS-PAGE gel for 4 h at 90 V and transferred to Immobilon-P polyvinylidene difluoride membranes (Merck Millipore) (Supplementary Figure S3B), in both cases by wet transfer [0.2 M glycine, 25 mM Tris, 20% (v/v) methanol] at 20 V overnight. Membranes were blocked in a blocking buffer [5% milk in Tris buffered saline and Tween 20 (TBST); 5% (w/v) non-fat powdered milk, 10 mM Tris–HCl (pH 7.6), 150 mM NaCl, 0.1% (v/v) Tween 20] for 1 h at RT followed by incubation with primary antibodies in 5% milk or 5% BSA in TBST overnight at 4°C and secondary antibodies (Supplementary Table S5) for 1 h at RT in dark. Bands were detected using a LI-COR Odyssey Imaging System (LI-COR Biosciences) (Figure 4B) or Amersham ECL and film exposure (Cytiva Amersham) (Supplementary Figure S3B).

### Colony formation assay

Cells (0.25 × 10^6^ cells/well) were seeded in six-well plates and treated with 16 nM palbociclib or DMSO for 24 h. Cells were harvested and 100 cells seeded per well in six-well plates in culture media, each in the absence or presence of palbociclib. Cells were incubated for 21 days, with media and drug replenished once a week. Colonies were stained with crystal violet [0.4% (w/v), Sigma] in 50% methanol.

### Statistical analysis

Statistical tests were performed using GraphPad Prism v8.4.0, except the Cox proportional-hazards model, which was implemented in RStudio v1.2.5033.

## DATA AVAILABILITY

Sequencing data and array CNV data are available through GEO: GSE168604. Flow cytometry data are available through FlowRepository: FR-FCM-Z5XQ. Code is available on GitHub (https://github.com/segondsa/resistant-colonies and https://github.com/s-andrews/celllinesleuth).

## Supplementary Material

zcac032_Supplemental_Files

## Notes

Present address: Prasanna Channathodiyil, Translational Medicine, Oncology R&D, AstraZeneca, Cambridge, UK.
