# Supplementary material for: Escape from G1 arrest during acute MEK inhibition drives the acquisition of drug resistance
Source: NAR Cancer. 2022 Oct 17;4(4):zcac032. doi: 10.1093/narcan/zcac032 (PMC9575185; doi:10.1093/narcan/zcac032)
Supplement: zcac032_Supplemental_Files [file zcac032_supplemental_files.zip › SI 6 revised (2)_PC edits.pdf]

Supplementary Figures

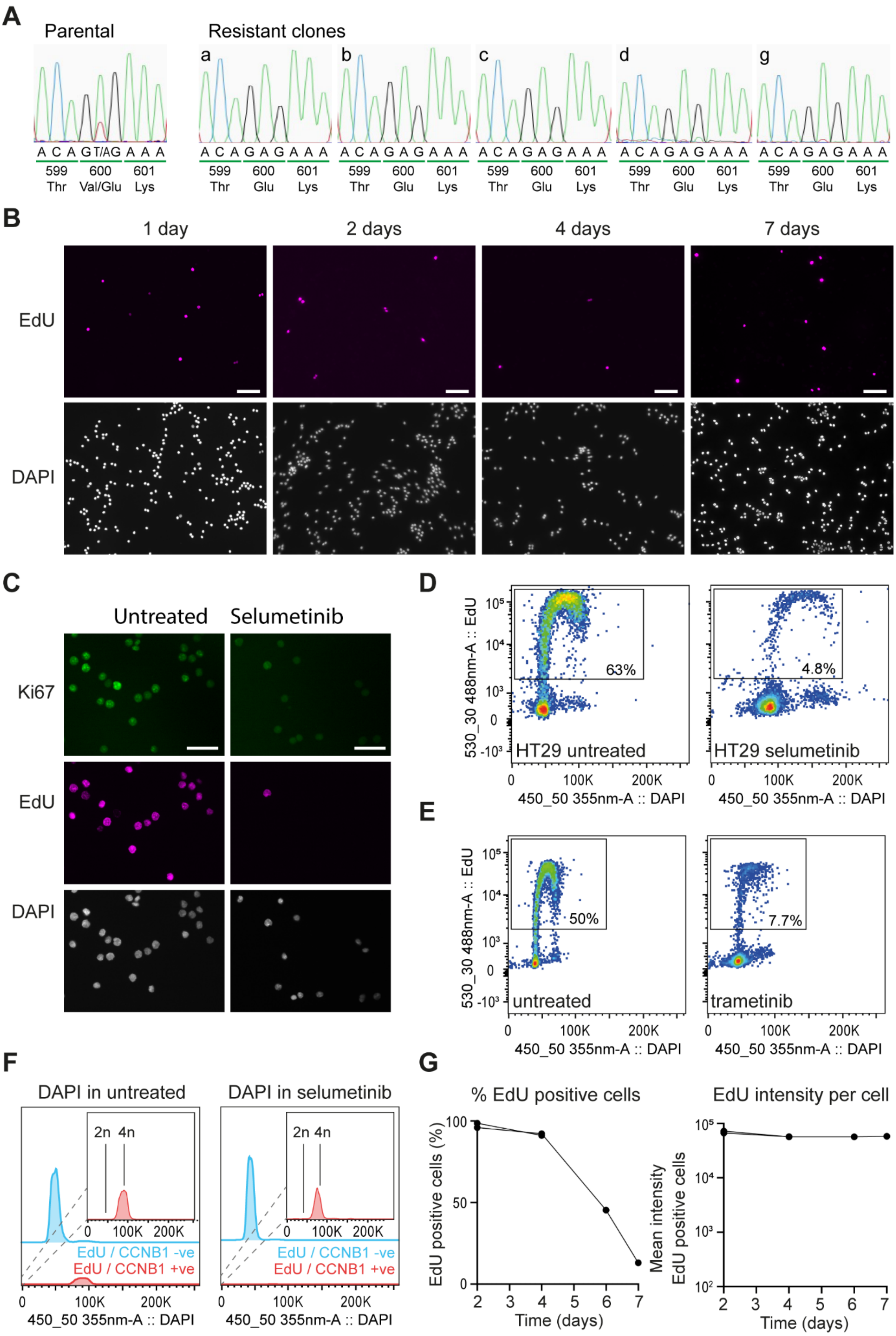

Figure S1: Supplement to replicating cells persist in long-term Selumetinib-treated cell cultures

A. Sanger sequencing traces for PCR products spanning the *BRAF*^{V600E} mutation in parental COLO205 cells and indicated selumetinib resistant clones from Figure 1B.

B. EdU incorporation in COLO205 cells treated with 1 µM selumetinib for the indicated duration before addition of 10 µM EdU for 24 hours in the presence of selumetinib. EdU positive cells (pink) co-stained with DAPI (grey) from selumetinib treated cells are shown (scale bars, 100 µm).

C. Ki67 staining in COLO205 cells treated with 1 µM selumetinib for 24 hours before addition of 10 µM EdU for 4 hours in the presence of selumetinib. For each channel - Ki67 staining (green), EdU (pink) and DAPI (grey) – images were acquired using the same exposure time and no differential post-processing applied between the untreated and treated images allowing semi-quantitative assessment of staining intensity (scale bars, 50 µm).

D. Quantification of EdU positive cells by flow cytometry in HT29 cells treated with 1 µM selumetinib or DMSO only (untreated) for 24 hours before addition of 10 µM EdU for 4 hours. Individual plots in B and C show EdU incorporation and DAPI staining of DNA for untreated (left) and selumetinib treated cells (right), with rectangles to indicate gates used to quantify EdU positive and negative cells.

E. Quantification of EdU positive cells by flow cytometry in clonal single-cell derivative (clone 1) of COL0205 cells treated with 3 nM trametinib or DMSO only (untreated) for 24 hours before addition of 10 µM EdU for 24 hours in the presence of 3 nM trametinib.

F. Fluorescence histograms of DAPI intensities for EdU / CCNB1 double negatives and double positives in untreated (left) and 1 µM selumetinib treated (right) COLO205 cells. Cells were treated with 1 µM selumetinib or DMSO only for 24 hours before addition of 10 µM EdU for 4 hours. EdU incorporation, CCNB1 and DAPI incorporation were determined by flow cytometry. Inset plots show DAPI intensities of EdU-CCNB1 double positives re-scaled to make rare EdU / CCNB1 double positive signals visible.

G. Quantification of EdU positive cells following a 24-hour EdU pulse during growth in the absence of selumetinib. COLO205 cells were treated with 2 µM EdU then washed with media and grown in culture for a period of 7 days. EdU incorporation was determined by flow cytometry on the indicated days, showing quantitation of EdU positive cells (left) and EdU intensity per cell (right), n=3.

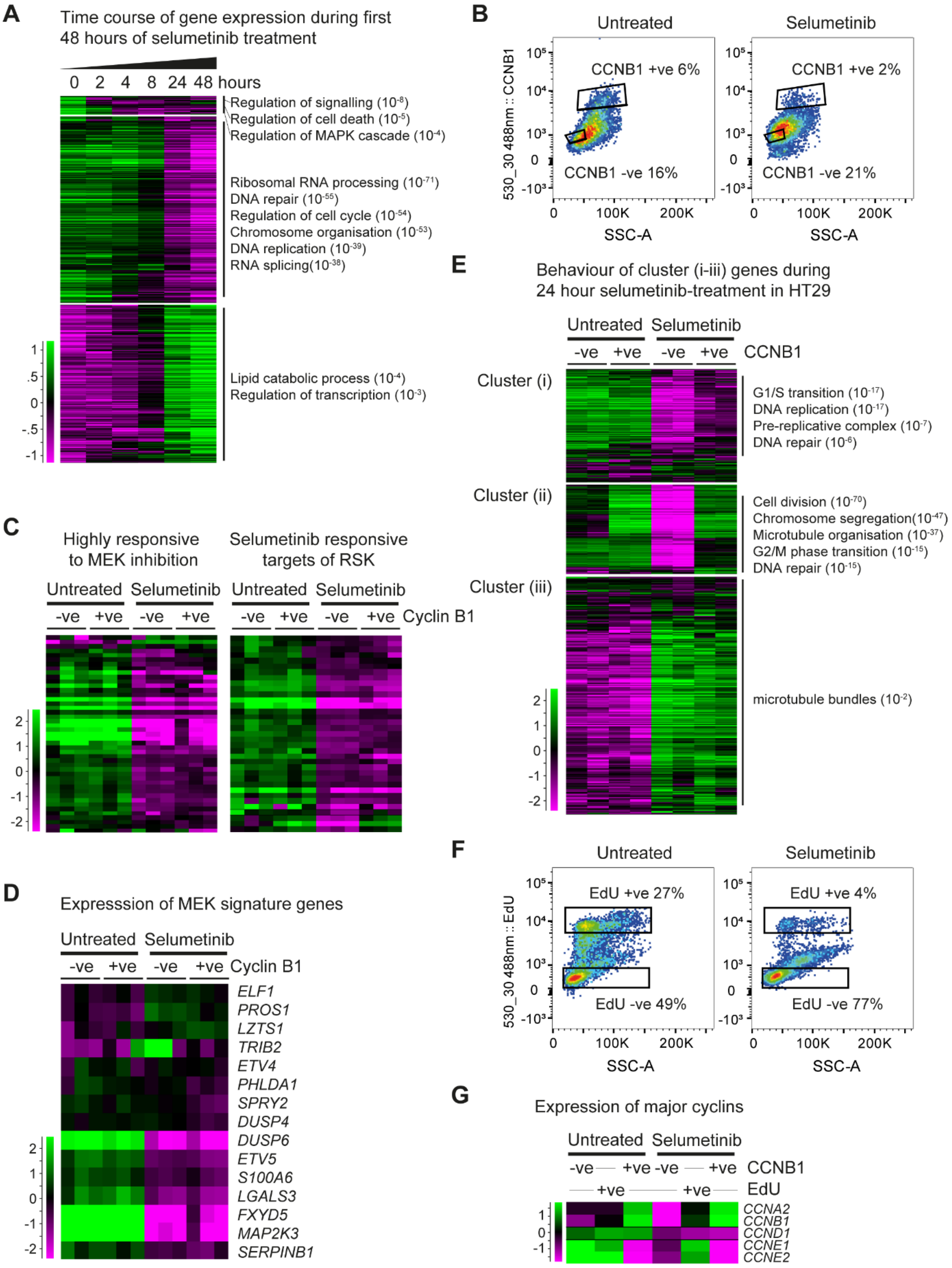
A
Time course of gene expression during first 48 hours of selumetinib treatment
0 2 4 8 24 48 hours
Regulation of signalling (10-8)
Regulation of cell death (10-5)
Regulation of MAPK cascade (10-4)
Ribosomal RNA processing (10-71)
DNA repair (10-55)
Regulation of cell cycle (10-54)
Chromosome organisation (10-53)
DNA replication (10-39)
RNA splicing(10-38)
Lipid catabolic process (10-4)
Regulation of transcription (10-3)
1
.5
0
-.5
-1
B
Untreated
Selumetinib
530_30 488nm :: CCNB1
CCNB1 +ve 6%
CCNB1 -ve 16%
CCNB1 +ve 2%
CCNB1 -ve 21%
SSC-A
0 100K 200K
C
Highly responsive to MEK inhibition
Selumetinib responsive targets of RSK
Untreated Selumetinib
-ve +ve -ve +ve Cyclin B1
2
1
0
-1
-2
D
Expresssion of MEK signature genes
Untreated Selumetinib
-ve +ve -ve +ve Cyclin B1
ELF1
PROS1
LZTS1
TRIB2
ETV4
PHLDA1
SPRY2
DUSP4
DUSP6
ETV5
S100A6
LGALS3
FXYD5
MAP2K3
SERPINB1
E
Behaviour of cluster (i-iii) genes during 24 hour selumetinib-treatment in HT29
Untreated Selumetinib
-ve +ve -ve +ve CCNB1
Cluster (i)
G1/S transition (10-17)
DNA replication (10-17)
Pre-replicative complex (10-7)
DNA repair (10-6)
Cluster (ii)
Cell division (10-70)
Chromosome segregation(10-47)
Microtubule organisation (10-37)
G2/M phase transition (10-15)
DNA repair (10-15)
Cluster (iii)
microtubule bundles (10-2)
F
Untreated
Selumetinib
530_30 488nm :: EdU
EdU +ve 27%
EdU -ve 49%
EdU +ve 4%
EdU -ve 77%
SSC-A
0 100K 200K
G
Expression of major cyclins
Untreated Selumetinib
-ve +ve -ve +ve CCNB1
+ve +ve EdU
CCNA2
CCNB1
CCND1
CCNE1
CCNE2

Figure S2: Supplement to gene expression analysis of replicating cells

A. Gene expression across time during selumetinib treatment in COLO205 cells treated with 1 µM selumetinib for up to 48 hours, with cultures harvested at indicated times. The 5782 genes significantly differentially expressed ($p<0.05$ by DEseq2) between 2 replicates of 0 and 48 hour time points are shown. Genes were categorised into 3 primary behaviours by hierarchical clustering, and representative enriched GO categories ($q<0.05$) are shown (full GO analysis is presented in Supplementary Table S5).

B. Flow cytometry density plots for COLO205 cells labelled with anti-CCNB1 primary antibody and donkey Alexa Fluor-488 conjugated secondary antibody and sorted using a BD FACSAria III sorter. Fluorescence thresholds for isolation of CCNB1 positive and negative cell fractions are shown. Gates were set based on a negative control staining without primary antibody, and the CCNB1 positive and negative sorting gates were set apart from each other to maximise sort purity.

C: (Left) Hierarchical clustering of gene set identified by Pratilas *et al*. (1) as significantly altered on MEK inhibition, showing relative expression in CCNB1 positive and negative fractions in the absence and presence of 1 µM selumetinib. (Right) Equivalent analysis for the subset of RSK targets identified by Doehn *et al*. (2) that decrease in expression on 1 µM selumetinib.

D. Hierarchical clustering of signature genes for MEK activity identified by Dry *et al.* (3) in CCNB1 positive and negative fractions in the absence and presence of 1 µM selumetinib.

E. Hierarchical clustering analysis of the three clusters of genes defined in Fig. 3B performed on HT29 cells either untreated or treated for 24 hours with 1 µM selumetinib and sorted for CCNB1, as in Fig. 3B. Full GO analysis is presented in Supplementary Table S6.

F. Flow cytometry density plots for COLO205 cells stained for EdU and sorted using a BD FACSAria III sorter. Fluorescence thresholds for isolation of EdU positive and negative cell fractions are shown. Gates were set using the unstained negative control and the EdU positive and negative sorting gates were set apart from each other to maximise sort purity.

G. Expression of the mRNAs encoding major cyclins extracted from the data in Fig. 3C.

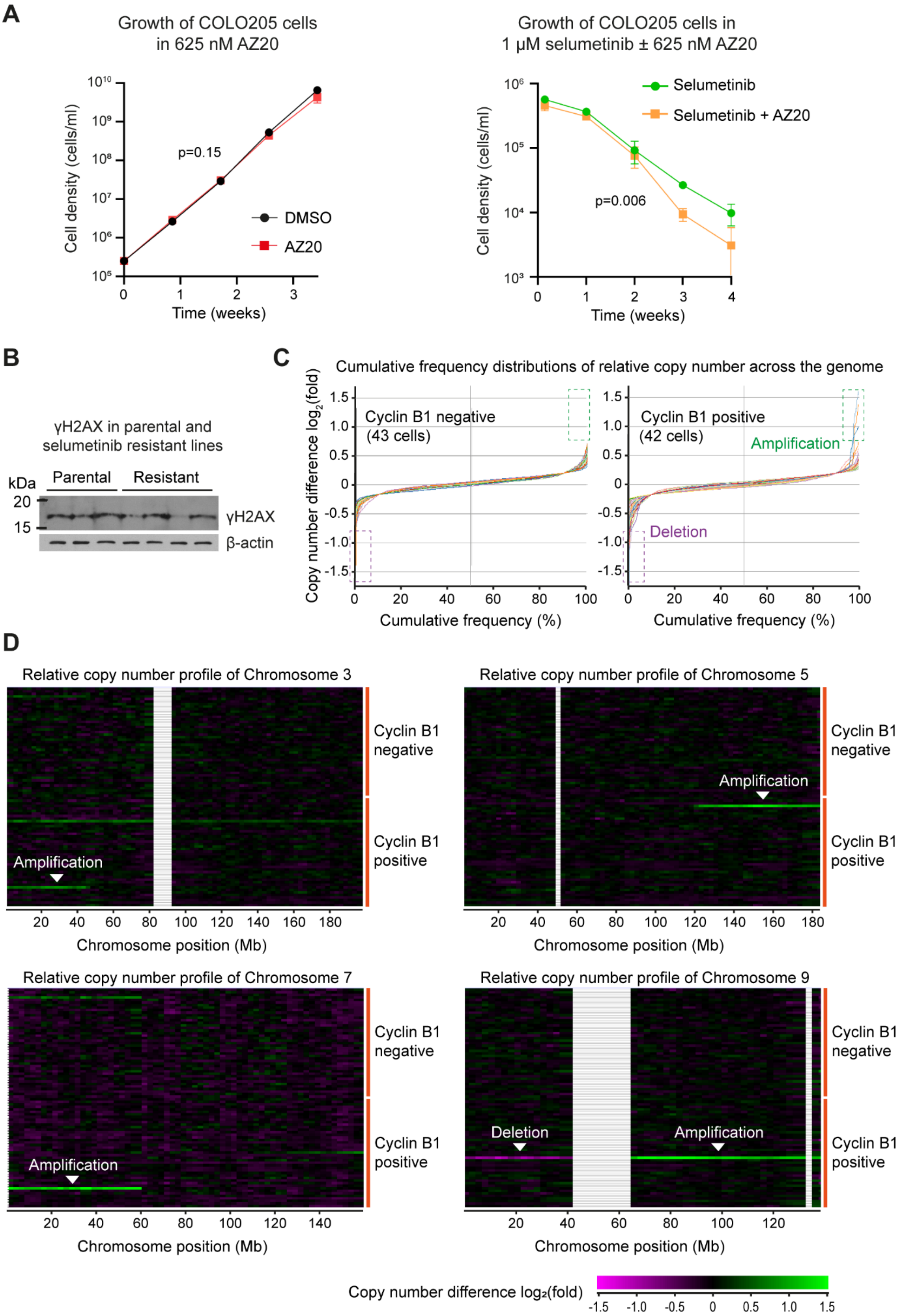

A
Growth of COLO205 cells in 625 nM AZ20
Cell density (cells/ml)
Time (weeks)
p=0.15
DMSO
AZ20
Growth of COLO205 cells in 1 μM selumetinib ± 625 nM AZ20
Selumetinib
Selumetinib + AZ20
p=0.006
B
γH2AX in parental and selumetinib resistant lines
kDa
Parental
Resistant
γH2AX
β-actin
C
Cumulative frequency distributions of relative copy number across the genome
Copy number difference log2(fold)
Cyclin B1 negative (43 cells)
Cyclin B1 positive (42 cells)
Amplification
Deletion
Cumulative frequency (%)
D
Relative copy number profile of Chromosome 3
Relative copy number profile of Chromosome 5
Relative copy number profile of Chromosome 7
Relative copy number profile of Chromosome 9
Cyclin B1 negative
Cyclin B1 positive
Amplification
Deletion
Chromosome position (Mb)
Copy number difference log2(fold)

Figure S3: Signs of replication stress during cell cycling in selumetinib

A. COL0205 cells were seeded at 0.25x10^6 cells/well in 6-well plates and treated with the indicated drugs (1 µM selumetinib and/or 625 nM AZ20) 24 hours later. Left: Every 6 days, cells were trypsinised, counted and 0.25x10^6 cells/well reseeded in media containing indicated drugs. Graph shows number of live cells at each time based on Trypan Blue staining, corrected for dilutions at each passage. Right: media and drug were replenished at weekly intervals without passaging, one well was harvested each week and live cells counted using Trypan Blue. All treatments were performed in parallel starting from the same cultures of cells, however we divided the data into two separate panels as the cells must be handled differently depending on whether they are proliferative (in the absence of selumetinib) or non-proliferative (in the presence of selumetinib). Error bars show SD, n=4 biological replicates for each condition, p values calculated by repeated measures two-way ANOVA.

B. Western blot for γH2AX and β-actin on total protein extracts of parental COLO205 cells (3 biological replicates) in the absence of drug, and selumetinib resistant cells cultured in 1 µM selumetinib (1 biological replicate each for 4 independent resistant lines).

C. Cumulative frequency plots showing the distribution of log-transformed copy number differences relative to parental genotype for all regions of the genome in 43 Cyclin B1 negative cells and 42 Cyclin B1 positive cells sorted from a population of COLO205 cells treated for 48 hours with 1 µM selumetinib. Probes showing amplification or deletion of 2 or more copies in a given cell (green and purple rectangles respectively) are likely to be bona fide copy number variation events.

D. Log-transformed copy number difference profiles for each of the 4 chromosomes where an extensive (10 Mb+) copy number amplification of 2 or more copies was detected. Each cell analysed is shown, segregated into Cyclin B1 negative and positive sets, amplified and deleted regions are indicated with white arrows. Underlying data is the same as in C, blank areas are low complexity regions that were excluded from the analysis.

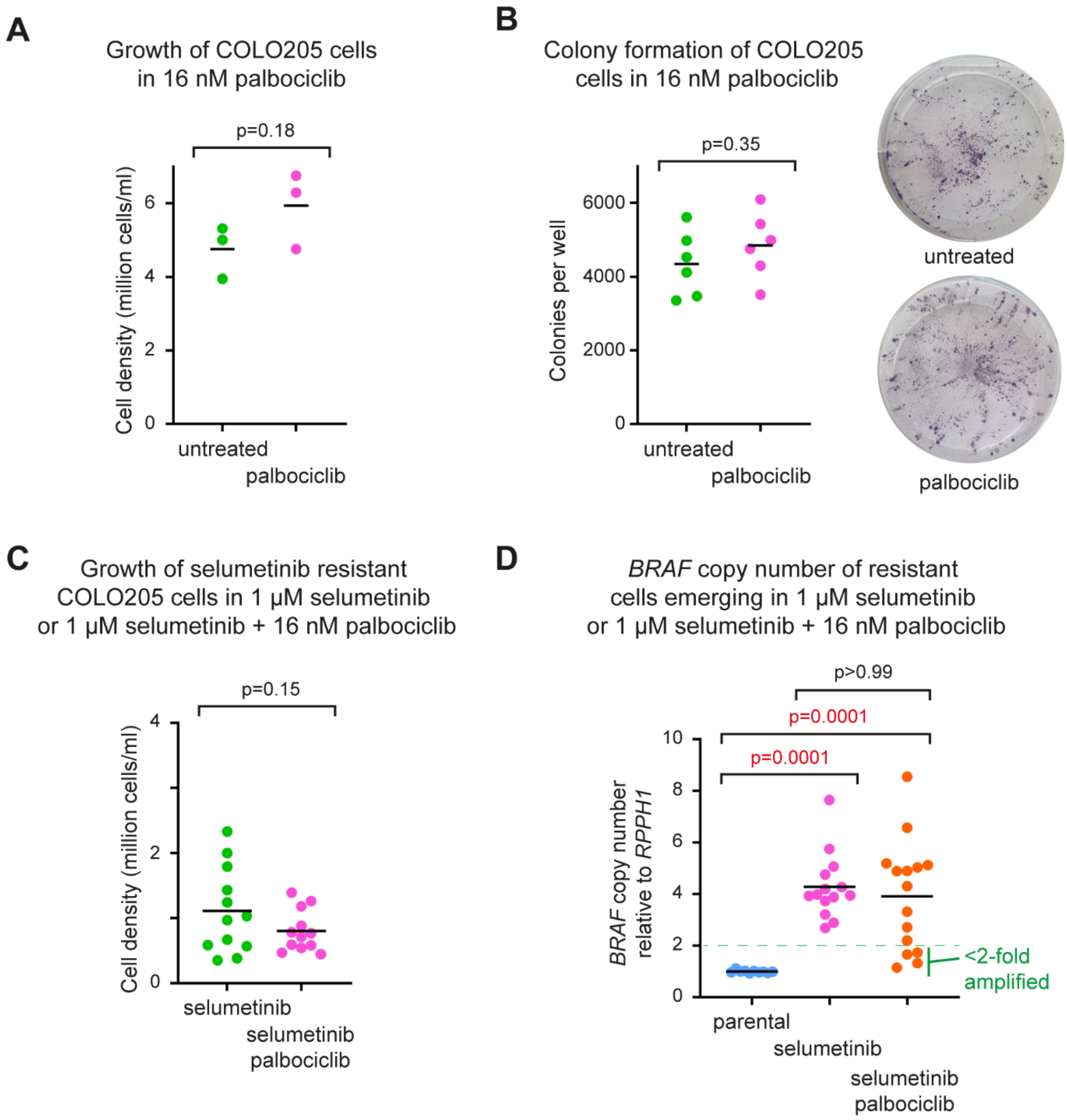
A
Growth of COLO205 cells
in 16 nM palbociclib
p=0.18
Cell density (million cells/ml)
0
2
4
6
untreated
palbociclib
B
Colony formation of COLO205
cells in 16 nM palbociclib
p=0.35
Colonies per well
0
2000
4000
6000
untreated
palbociclib
untreated
palbociclib
C
Growth of selumetinib resistant
COLO205 cells in 1 μM selumetinib
or 1 μM selumetinib + 16 nM palbociclib
p=0.15
Cell density (million cells/ml)
0
2
4
selumetinib
selumetinib
palbociclib
D
BRAF copy number of resistant
cells emerging in 1 μM selumetinib
or 1 μM selumetinib + 16 nM palbociclib
p>0.99
p=0.0001
p=0.0001
BRAF copy number
relative to RPPH1
0
2
4
6
8
10
<2-fold
amplified
parental
selumetinib
selumetinib
palbociclib

Figure S4: Second supplement to suppressing DNA replication in selumetinib slows acquisition of resistance

A. COLO205 cells were seeded at 0.25×10^6 cells/well in 6-well plates and treated with 16 nM palbociclib or DMSO only for 24 hours, after which 100 cells from each condition were re-plated in media containing 16 nM palbociclib or DMSO only and cultured for 2 weeks with media and drug replenished weekly and counted using a Countess automated cell counter at the end of 2 weeks. p value calculated by t test, n=3 biological replicates.

B. COLO205 cells were treated and re-plated at 100 cells per well as in B to allow formation of colonies, then fixed and stained in 0.4% crystal violet in 50% methanol (representative images shown). Number of colonies was quantified, p value calculated by t test, n=6 biological replicates per condition.

C. Cell counts determined for selumetinib resistant cells derived from single-cell derivative of COLO205 cells (clone 2) as in B in the presence of 1 µM selumetinib. p value calculated by t test, n=12 biological replicates.

D: qPCR copy number analysis of *BRAF* relative to control gene *RPPH1* in parental and selumetinib-resistant cell lines derived in selumetinib alone or selumetinib + palbociclib. COLO205 cells were treated with 1 µM selumetinib in the presence and absence of 16 nM palbociclib, and media and drug changed weekly until colonies of proliferating cells were observed. Each sample was assayed in triplicate. p values were calculated by Kruskal-Wallis test (n=10 parental, 14 selumetinib, 15 selumetinib + palbociclib). The subset of resistant clones with <2-fold *BRAF* amplification are indicated.

Supplementary Tables

Antibodies	Supplier	Catalogue	Dilution
**AlexaFluor 488 donkey anti-rabbit antibody**	ThermoFisher Scientific	#A21206	1:1000 (IF)
**AlexaFluor 594 donkey anti-rabbit antibody**	ThermoFisher Scientific	#A11037	1:1000 (FC)
**Anti-rabbit IgG (H+L) (DyLight 800 4x PEG Conjugate)**	LI-COR	#5151	1:30000 (WB)
**Anti-mouse IgG (H+L) (DyLight 800 4x PEG Conjugate)**	LI-COR	#5257	1:30000 (WB)
**CCNB1; rabbit monoclonal (clone D5C10)**	CST	#12231	1:200 (FC)
**CCND1 (Cyclin D1); mouse monoclonal (clone DCS-6)**	Merck Millipore	#CC12	1:200 (WB)
**GAPDH; rabbit monoclonal (clone EPR16891)**	Abcam	#ab181602	1:2000 (WB)
**Phospho-p44/42 MAPK (Erk1/2) (Thr202/Tyr204); rabbit monoclonal (clone D13.14.4E)**	CST	#4370	1:400 (IF)
**p27KIP1; mouse monoclonal (clone DCS72)**	Merck Millipore	#NA35	1:200 (WB)
**Phospho-Rb S795; rabbit polyclonal**	CST	#9301	1:500 (WB)
**RB; mouse monoclonal (clone 4H1)**	CST	#9309	1:1000 (WB)
**β-Actin; mouse monoclonal**	Sigma-Aldrich	#A5441	1:10000 (WB)
**Phospho-γH2AX S139; mouse monoclonal**	CST	#80312	1:500 (WB)
**Ki67; Rat monoclonal, FITC conjugated**	Invitrogen	11-5698-82	1:200 (IF)

Supplementary Table S1. List of antibodies used in this study

Gene name	Primer sequence
BRAF	Forward 5'-TCTTGTAACATCCTTTATAGCAAACCAGT-3'
	Reverse 5'-GCTATAAATCAAGGATTCCCACATCTTCT-3'
CFTR	Forward 5'-TAGGAAGTCACCAAAGCAGTACAGC-3'
	Forward 5'-AGCTATTCTCATCTGCATTCCAATG-3'
RPPH1	Forward 5'-AGATTTGGACCTGCGAGCG-3'
	Reverse 5'-GAGCGGCTGTCTCCACAAGT-3'
BRAF	Forward 5'-GCCCCAAAAATCTTAAAAGCAGGTT-3'
spans V600	Reverse 5'-TGAAATACACTGAAACTGGTTTCAAAATATTCGT-3'

Supplementary Table S2. List of PCR primers used in this study

Supplementary Tables S3-S6 provided as separate Excel files.

References

1. C. A. Pratilas *et al.*, (V600E)BRAF is associated with disabled feedback inhibition of RAF-MEK signaling and elevated transcriptional output of the pathway. *Proc Natl Acad Sci U S A* **106**, 4519-4524 (2009).
2. U. Doehn *et al.*, RSK is a principal effector of the RAS-ERK pathway for eliciting a coordinate promotile/invasive gene program and phenotype in epithelial cells. *Mol Cell* **35**, 511-522 (2009).
3. J. R. Dry *et al.*, Transcriptional pathway signatures predict MEK addiction and response to selumetinib (AZD6244). *Cancer Res* **70**, 2264-2273 (2010).
